# *Angelica gigas* Nakai and Soluplus-Based Solid Formulations Prepared by Hot-Melting Extrusion: Oral Absorption Enhancing and Memory Ameliorating Effects

**DOI:** 10.1371/journal.pone.0124447

**Published:** 2015-04-27

**Authors:** Jingpei Piao, Jae-Young Lee, Jin Bae Weon, Choong Je Ma, Hyun-Jeong Ko, Dae-Duk Kim, Wie-Soo Kang, Hyun-Jong Cho

**Affiliations:** 1 School of Bioscience and Biotechnology, Kangwon National University, Chuncheon, 200–701, Republic of Korea; 2 College of Pharmacy and Research Institute of Pharmaceutical Sciences, Seoul National University, Seoul, 151–742, Republic of Korea; 3 Department of Medical Biomaterials Engineering, College of Biomedical science, Kangwon National University, Chuncheon, 200–701, Republic of Korea; 4 Research Institute of Biotechnology, Kangwon National University, Chuncheon, 200–701, Republic of Korea; 5 College of Pharmacy, Kangwon National University, Chuncheon, 200–701, Republic of Korea; Texas Tech Univ School of Pharmacy, UNITED STATES

## Abstract

Oral solid formulations based on *Angelica gigas* Nakai (AGN) and Soluplus were prepared by the hot-melting extrusion (HME) method. AGN was pulverized into coarse and ultrafine particles, and their particle size and morphology were investigated. Ultrafine AGN particles were used in the HME process with high shear to produce AGN-based formulations. In simulated gastrointestinal fluids (pH 1.2 and pH 6.8) and water, significantly higher amounts of the major active components of AGN, decursin (D) and decursinol angelate (DA), were extracted from the HME-processed AGN/Soluplus (F8) group than the AGN EtOH extract (ext) group (*p* < 0.05). Based on an *in vivo* pharmacokinetic study in rats, the relative oral bioavailability of decursinol (DOH), a hepatic metabolite of D and DA, in F8-administered mice was 8.75-fold higher than in AGN EtOH ext-treated group. In scopolamine-induced memory-impaired mice, F8 exhibited a more potent cognitive enhancing effect than AGN EtOH ext in both a Morris water maze test and a passive avoidance test. These findings suggest that HME-processed AGN/Soluplus formulation (F8) could be a promising therapeutic candidate for memory impairment.

## Introduction

*Angelica gigas* (Dang-Gui) is a biennial or short lived perennial plant found in China, Japan, and Korea. The root of *Angelica gigas* has been used in oriental traditional medicine and is marketed as a functional food product in Europe and North America [1]. Cham-Dang-Gui (Korean Angelica, the dried root of *Angelica gigas* Nakai (AGN)) has been principally cultivated in Korea and used as a Korean medicinal herb. It contains several chemicals, such as pyranocoumarins, essential oils, and polyacetylenes [2]. The major active components of AGN are hydrophobic pyranocoumarins, including decursin (D), decursinol (DOH), and decursinol angelate (DA). They have analgesic, anticancer, anti-inflammatory, and neuroprotective effects [2–5]. Particularly, the active ingredients of AGN, as single components or extracts, have been reported to ameliorate memory impairment in animal studies [6,7]. Furthermore, the pharmacological efficacies and *in vivo* pharmacokinetic properties (including absorption and metabolism) after oral administration of the active components of AGN have been demonstrated [8,9].

Natural products can be processed by extraction, filtration, concentration, freeze drying, and their combination, but these methods are generally time-consuming, labor-intensive, and expensive. For the efficient extraction of pharmacologically active components, alcohols have generally been used as extraction solvents [10]. Besides alcohols, hot water can be used to extract bioactive components from AGN. However, that method seems to be inappropriate for extracting poorly water-soluble components from AGN [11]. Several major components of AGN, such as D and DA, are reported as poorly water-soluble, thus improving the aqueous solubility of those active components is necessary.

Diverse approaches have been applied to enhance aqueous solubility and dissolution of poorly water-soluble components, including chemical modification, physical modification, and carrier systems [12–14]. Among them, the hot-melting extrusion (HME) technique has been used to prepare solid formulations (i.e. solid dispersion) of poorly water-soluble components [15]. It offers several advantages over the other methods: HME is fast, continuous, environmentally friendly, and economical [16–18].

Using the HME technique, the active compounds of natural products can be expected to disperse at a molecular level, thereby improving their aqueous solubility and/or dissolution rate and prolonging their storage life. Recently, the particle size of orally administered AGN powders has been reported to influence the treatment of estrogen-related symptoms of menopause [11]. Compared to coarse AGN powder, ultrafine AGN powder exhibited superior pharmacological efficacies in terms of serum ovarian and reproductive hormone levels and experimental osteoporosis parameters. Increasing the powder’s surface area to volume ratio by ultrafine milling seems to increase the dissolution of active components and subsequently enhance the bioactivity of herbal medicines. This milling method can also be used for extracts, which are likely to lose their bioactivity during processing, storage, and oral intake [11].

Herein, two methods including physical modification (milling and HME) and chemical modification (the addition of Soluplus) were introduced to prepare oral solid formulations of AGN. Ultrafine milled AGN particles with Soluplus were processed by HME to increase the aqueous solubility of pharmacologically active components (D and DA) of AGN. Soluplus, a grafted copolymer composed of polyethylene glycol (PEG) 6000, vinylcaprolactam, and vinyl acetate has been reported as a suitable polymer for HME processing [19,20]. It is known to enhance the aqueous solubility, dissolution, and absorption of poorly water-soluble drug [21–22]. In this study we performed physicochemical characterization and extraction of developed AGN-based solid formulations and assessed their pharmacokinetic properties and pharmacological efficacy *in vivo*.

## Methods and Materials

### Materials

Fresh *Angelica gigas* Nakai (AGN) was purchased from Pyeongchang (Korea). Standard samples of D, DA, and DOH were obtained from Korea Promotion Institute for Traditional Medicine Industry (Gyeongsan, Korea). Soluplus was purchased from BASF (Ludwigshafen, Germany). Carboxymethyl cellulose sodium (CMC-Na) was obtained from Sigma-Aldrich Co. (St. Louis, MO, USA). All solvents used in this study were high performance liquid chromatography (HPLC) grade. All other chemicals were of analytical grade and used without further purification.

### Preparation and characterization of AGN formulations

AGN was dried in the oven at 55°C for 24 h and cooled at room temperature. The AGN sample was then stored at 4°C until milling. Coarse and ultrafine powder formulations were acquired by the reported milling methods with slight modifications [11]. AGN samples were milled into coarse powder by a pin crusher (JIC-P10-2; Myungsung Machine, Seoul, Korea) equipped with a 30-mesh sieve. The milled powder was fractionated using a sieve shaker (CG-213, Ro-Top, Chunggye Industrial Mfg. Co., Seoul, Korea) equipped with a series of sieves (Φ 20 cm). The powder was passed through 300-μm mesh size sieves, and unpassed particles were grinded again with the pin crusher. Those powders were then stored at 25°C before ultrafine milling. The coarse powders were pulverized and classified by a low temperature turbo mill (HKP-05; Korea Energy Technology Co., Ltd., Seoul, Korea). The powders were pulverized after passing an impeller with high rotation speed, and the first, second, and third stator classifier system was used to classify particles using centrifugal and drag forces. For ultrafine powder processing, the rotor speed was set as 10,500 rpm, and the temperature of mill chamber was kept at -18°C. The ultrafine AGN powder obtained was stored in a desiccator before its use.

Particle sizes of coarse and ultrafine AGN (F1 and F2) were measured by a particle size analyzer (Mastersizer 2000; Malvern Instruments Ltd., Worcestershire, UK), by using the laser diffraction technique. Particle size was measured at 25°C with a scattering angle of 90°. The average particle size indicates the mean value of 9 measurements for every sample.

AGN-based oral formulations were prepared in this investigation according to the experimental conditions and composition ratios shown in Table 1. For solid formulations, ultrafine AGN powder and water (4:1, w/w) was extruded by different shears using an STS-25HS twin-screw extruder (Hankook E.M. Ltd., Pyoung-Taek, Korea) equipped with a round-shaped die (1 mm in diameter), according to the presented temperatures (Table 2). Three different (low, middle, and high) shear stresses were generated by the different screw arrangements. Each shear stress (low, middle, and high) was represented by measuring specific mechanical energy (SME) [23]. The feeding amount of sample was 28 g. Water (20% of total input weight) was added at 1.0 mL/min speed to the extruder. The speed of screw was 150 rpm and the diameter of die was 1.0 mm, respectively. In Soluplus-included formulations, ultrafine AGN powder was mixed with the determined ratios of Soluplus and then extruded with high shear stress. Prepared AGN-based formulations were dried in the oven at 40°C.

**Table 1:** The compositions and processing conditions of developed formulations.

Formulations	AGN (%)	Soluplus (%)	Grinding degree	Shear stress
**F1**	100	0	coarse	-
**F2**	100	0	ultrafine	-
**F3**	100	0	ultrafine	low
**F4**	100	0	ultrafine	middle
**F5**	100	0	ultrafine	high
**F6**	90	10	ultrafine	high
**F7**	70	30	ultrafine	high
**F8**	50	50	ultrafine	high

**Table 2:** Temperature (°C) profile in the barrel sections of the hot-melting extruder.

Formulations	Barrel section							
	1	2	3	4	5	6	7	Die
**F3-F5**	-	-	80	80	90	100	-	-
**F6-F8**	-	-	90	90	100	100	130	130

The speed of screw was 150 rpm.

### Water absorption studies

Water absorption-related indexes were determined in triplicate, according to the reported method with slight modifications [24]. One gram of sample was suspended in 30 mL of DW at room temperature, gently stirred for 1 h, and then centrifuged at 3,000 rpm for 20 min. The supernatant was decanted into an evaporating dish of known weight. Related parameters, such as water absorption index (WAI), water solubility (WS), and swelling power (SP) were calculated by the following formulas:

$$\text{WAI}=\frac{\text{the weight of wet sediment}}{\text{the weight of dry sample}}$$ (1)

$$\text{WS (\%)}=\frac{\text{the weight of dried supernatant}}{\text{the weight of dry sample}}\times 100$$ (2)

$$\text{SP}=\frac{\text{the weight of wet sediment}}{\text{the weight of dry sample }\times \text{ (1-}\frac{\text{WS (\%)}}{\text{100}}\text{)}}$$ (3)

### Particle size analysis of aqueous dispersion of formulations

Each developed AGN formulation (0.3 g) was suspended in 30 mL of DW, and the supernatant was separated by centrifugation at 3,000 rpm for 20 min. The particle-related properties of the supernatant (particle size and polydispersity index) were studied using a light-scattering spectrophotometer (ELS-Z1000; Otsuka Electronics, Tokyo, Japan).

### Determination of major components in AGN EtOH extract

The content of D, DA, and DOH in the AGN EtOH extract (ext) was determined by liquid chromatography-tandem mass (LC-MS/MS) system. An aliquot (5 μL) of AGN EtOH-ext dissolved in methanol (1 μg/mL) was injected onto LC-MS/MS system equipped with an Agilent Technologies 1260 Infinity HPLC system (Agilent Technologies, Wilmington, DE, USA) and Agilent Technologies 6430 Triple Quad LC/MS system. The chromatographic separation of D and DA was achieved by using XTerra MS C18 3.5 μm column (150 × 2.1 mm; Waters, MA, USA) with a C18 guard column (4 × 2.0 mm; Phenomenex, CA, USA). The mobile phase consisted of acetonitrile (A) and 5 mM ammonium formate buffer (B) at a flow rate of 0.2 mL/min. The gradient elution program used was as follows: (1) The ratio of mobile phase A was set at 10% and maintained for 1 min, (2) a linear gradient method was run until it became 70% in 45 min, (3) a linear gradient was run back to make it 10% in 0.1 min and maintained until the pump pressure returned to the initial value. The total run time was 55 min. The fragmentation transitions of D and DA were identical to each other: *m/z* 329.2 to 229.1. The fragmentor voltage and collision energy were 130 V and 18 eV, respectively. The retention times of D and DA were 38.54 and 38.85 min, respectively. An aliquot (5 μL) of AGN EtOH ext dissolved in methanol (10 μg/mL) was injected onto LC-MS/MS system for DOH analysis. Chromatographic separation of DOH was achieved by using a Synergi 4 μ Hydro-RP 80 Å column (75 × 2.0 mm; Phenomenex, CA, USA). The mobile phase was composed of acetonitrile and 5 mM ammonium formate buffer (70:30, v/v), and the flow rate was 0.4 mL/min. The fragmentation transitions for DOH was *m/z* 247.1 to 229.1. The retention time of DOH was 0.71 min. The ESI source settings for the analysis of D, DA, and DOH were optimized manually; gas temperature, gas flow, nebulizer pressure, and capillary voltage were 300°C, 11 L/min, 15 psi, and 4000 V, respectively. The acquisition and processing were performed with MassHunter Workstation Software Quantitative Analysis (Version B.05.00; Agilent Technologies). Linearity was established in the range of 20–1,000 ng/mL of D and DA concentration. Precision and accuracy of both components were within the acceptable ranges.

### Extraction test

Each sample of AGN formulation (0.3 g) was added to 30 mL of DW, pH 1.2 buffer, and pH 6.8 buffer, and incubated in the shaker at 40°C for 2 h. Next, the mixtures were filtered to separate the supernatant and sediment, and the supernatants were dried. The amounts of D and DA in those samples were quantitatively analyzed by a high performance liquid chromatography (HPLC) system (CBM-20A, Shimadzu Co, Ltd., Tokyo, Japan) equipped with a pump (LC-20AT, Shimadzu), an autosampler injector (SIL-20A, Shimadzu), a UV/Vis detector (SPD-10A, Shimadzu), and a column oven (CTO-20A, Shimadzu) set at 35°C. A C18 column (Kinetex, 100 × 4.6 mm, 2.6 micron, Phenomenex, Torrance, CA, USA) was used, and the flow rate was 1.0 mL/min. The mobile phase was composed of solvent A (0.4% formic acid in water) and solvent B (acetonitrile), and a gradient mode was used (0–15 min, 33–45% solvent B; 15–30 min, 45–55% solvent B; 30–40 min, 55–80% solvent B; 40–45 min, 80–33% solvent B). The injection volume was 10 μL, and the eluent was monitored at 329 nm. Linearity was established in the range of 10–1,000 μg/mL D or DA concentration. Precision and accuracy of both components were within the acceptable ranges.

### *In vivo* pharmacokinetic study

The *in vivo* pharmacokinetic properties of DOH after oral administration of AGN-based formulations were studied in male Sprague-Dawley (SD) rats (250 ± 5 g of body weight; Orient Bio, Sungnam, Korea). The rats were reared in a light-controlled room at 22 ± 2°C and at 55 ± 5% relative humidity. The experimental protocols of animal studies were approved by the Animal Care and Use Committee of the College of Pharmacy (Seoul National University, Seoul, Korea).

The left femoral artery was cannulated with an Intramedic polyethylene tube (PE-50; Becton Dickinson Diagnostics, MD, USA) under 50 mg/kg of Zoletil (intramuscular injection; Virbac, Carros, France) anesthesia. Each formulation was suspended in DW and administered orally at doses corresponding to 100 mg/kg EtOH ext of AGN. Corresponding dose of each formulation was determined based on the sum of D and DA contents in AGN EtOH ext, analyzed by described LC-MS/MS method. Blood samples (200 μL) were collected from the femoral artery at determined times (5, 15, 30, 60, 90, 120, 240, and 480 min), and the equivalent volume of normal saline (containing 20 U/mL heparin) was supplemented at each sampling time. Blood samples were centrifuged at 16,000 × g at 4°C for 3 min, and aliquots (70 μL) of supernatant were stored at -70°C before analysis.

DOH concentration in rat plasma was determined using a LC-MS/MS system as described in previous section with slight modification. Losartan (5 μL, LST, internal standard) solution (10 μg/mL) and 95 μL of acetonitrile were added to a 50 μL aliquot of plasma sample and mixed for 5 min. After centrifugation at 16,000 × g for 5 min, the aliquot (5 μL) of supernatant was injected into the LC-MS/MS system equipped with an Agilent Technologies 1260 Infinity HPLC (Agilent Technologies) and Agilent Technologies 6430 Triple Quad LC/MS system. The extraction recovery of DOH from plasma samples was 96.50 ± 2.58%. The LC and MS conditions for DOH analysis were same with described method. The fragmentation transition for LST was *m/z* 423.4 to 207.3. The fragmentor voltage and collision energy were 115 V and 20 eV for LST. The retention time of LST was 0.47 min. Acquisition and processing were performed with MassHunter Workstation Software Quantitative Analysis (Version B.05.00; Agilent Technologies). Linearity was established in the range of 2–10,000 ng/mL DOH concentration. Lower limit of quantitation (LLOQ) was 2 ng/mL. Precision was within 5.9%, and accuracy was ranged from -7.0 to 10.5% in this analysis method, respectively.

The following pharmacokinetic parameters of DOH were calculated by WinNonlin (Version 3.1; Pharsight, Mountain View, CA, USA): total area under the plasma DOH concentration–time curve from time zero to time infinity (AUC), maximum concentration (C_max_), and the time of maximum concentration observed (T_max_).

### Histological study

The toxicity of developed formulations on the intestinal epithelium was evaluated by histological assay. EtOH ext of AGN and AGN-loaded formulations (F2, F5, and F8) were orally administered to SD rats at a dose of 100 mg/kg (AGN EtOH ext), and rats were euthanized 24 h post-administration. The jejunum was dissected and fixed in 10% (v/v) formaldehyde solution for 1 day. Tissues were rinsed with tap water, dehydrated with alcohols, and embedded in paraffin. Tissues were then cut into 5–10 μm thick sections and stained with hematoxylin and eosin (H&E) reagent. Microscopic images were taken to assess the mucosal toxicity of the developed formulations.

### *In vivo* memory and learning tests

Imprinting control region (ICR) mice (male, 4-week-old, 25–30 g of body weight) were purchased from Daehan Biolink Co., Ltd. (Chungbuk, Korea) and reared at 20 ± 3°C under a 12/12-h light-dark cycle with access to food (commercial pellet) and water *ad libitum*. All animal experimental procedures were approved by the Institutional Animal Care and Use Committee (IACUC) of Kangwon National University (KIACUC).

AGN EtOH ext, F8, and donepezil (1 mg/kg) were dissolved in 0.5% CMC-Na solution, and scopolamine was dissolved in normal saline (0.9% NaCl). In case of AGN EtOH ext and F8-treated groups, oral doses were corresponded to 200 mg/kg of AGN EtOH ext. As described, each dose was based on the contents of the sum of D and DA. Both control and scopolamine alone treated groups received 0.5% CMC-Na solution orally. The positive control group was administered donepezil orally. Following 90 min of AGN EtOH ext and F8 administrations, scopolamine was subcutaneously administered in four groups, excluding the control group. Trials of the Morris water maze test and passive avoidance test were performed 30 min after scopolamine administration.

The Morris water maze test was performed based on previous reports with slight modifications [25,26]. The water maze consisted of large circular pool (90 cm in diameter and 40 cm in height) filled with opaque water (20 ± 1°C) to a depth of 30 cm by using white milk. The water maze was divided into four equal quadrants. A white escape platform was submerged in one of the pool quadrants, and its location was fixed across 4 days. The same quadrants were not used for the starting point during the test trials. All swimming activity was monitored and recorded using a video camera linked to a smart video-tracking system. The time to reach the platform was recorded as the escape latency. If mice did not find the platform within 120 s, they were guided to the platform, kept there for 10 s, and the escape latency was recorded as 120 s. On the last day, a probe trail test was done by removing the platform for 60 s. The swimming time in the quadrant where the platform was placed was recorded to evaluate the memory function.

The passive avoidance test was performed according to previously reported methods [25,26]. A passive avoidance apparatus (Gemini system, San Francisco, CA, USA) has two compartments (17 cm × 12 cm × 10 cm) with an electrifiable grid floor separated by a guillotine door. The drug administration protocol for this test was identical to that used for the Morris water maze test. The training trial was performed on the first day. When the mice completely moved from the light to dark compartment after guillotine door opening, the door was closed automatically, and a 2 s electric foot-shock (0.1 mA/10 g body weight) was delivered through the grid floor. The test trial began 24 h after the training trial, and the elapsed time before mice entered the dark compartment (latency time) was measured. If a mouse waited more than 180 s in the light compartment, then it was excluded from this experiment.

### Statistical analysis

All experiments in this investigation were performed at least three times, and the data were presented as the mean ± standard deviation (SD). Statistical analyses were done by two-tailed Student’s *t*-test or analysis of variance (ANOVA). *p* values less than 0.05 indicate a statistically significant difference.

## Results and Discussion

### Preparation and characterization of AGN-based formulations

**Fig 1:**
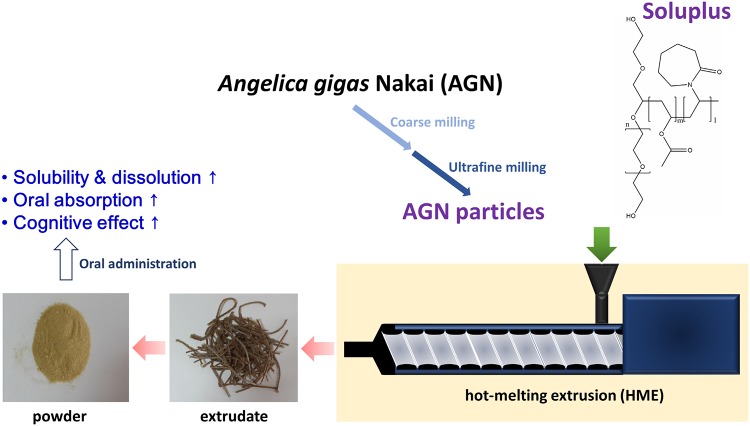
Scheme of developing AGN-based solid formulations by HME technique.

AGN-based oral formulations were prepared using the HME technique (Fig 1). Prior to the HME process, AGN was pulverized into ultrafine particles. Different shear stress and weight ratios of Soluplus were tested to optimize the HME process (Table 1). Soluplus was included as a polymer matrix for oral solid formulations of AGN, and the temperatures in the barrel section were set as presented in Table 2 during the HME process. Since the glass transition temperature (T_g_) of Soluplus is around 70°C and the heating zone of barrel can be set 15–60°C above the T_g_ of the polymer [16], established temperatures in the barrel section seem to be appropriate.

The degree of shear stress can be explained by SME. SME can be defined as the consumption of mechanical energy during extrusion process per mass flow rate [23]. It is dependent on the conditions of extrusion process such as screw speed, barrel temperature, water content, feed composition, and screw configuration. Different screw configuration used in this study can produce different degree SME values. As shown in Table 3, SME value was increased in the order of low < middle < high shear stress.

**Table 3:** Specific mechanical energy (SME) of each shear stress condition.

Shear stress	SME (Wh/kg)
**Low**	41.52 ± 3.18
**Middle**	65.80 ± 3.59
**High**	79.15 ± 9.57

SME was calculated based on slight modified formula presented in the reference [23].

$$\text{SME}\;=\;\frac{\text{Mechanical energy input}}{\text{Mass flow rate}}\;=\;\frac{N_{act}}{N_{max}}\;\times \;\frac{\tau_{act}}{\tau_{max}}\;\times \;\frac{K_{w}}{\text{Q}}$$

N_act_: actual screw speed (rpm)

N_max_: maximum screw speed (408.74 rpm in this study)

τ_act_: actual torque value (N∙m)

τ_max_: maximum torque value (175.16 N∙m in this study)

K_w_: power of direct current motor (7.5 kW)

Q: the amount of product per time (kg/h)

**Fig 2:**
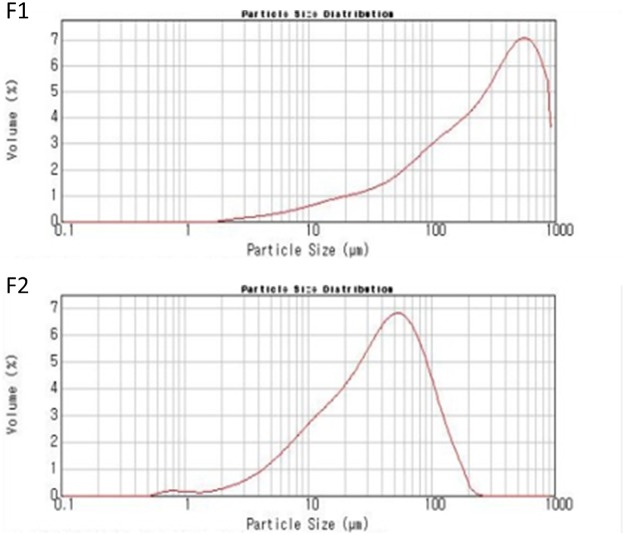
Size distribution of coarse and ultrafine AGN particles (F1 and F2).

To put AGN into the HME machine, the particle size of AGN powder was reduced. After coarse milling, the mean diameter of particles (F1) was 329.3 ± 2.4 μm (Table 4). To further reduce particle size, coarse powders (F1) were milled into ultrafine particles (F2), which have a mean diameter of 47.9 ± 2.5 μm (Table 4). Particle size reduction, from coarse to ultrafine, by milling processes is shown in the particle size distribution chart (Fig 2). The narrow size distribution of coarse and ultrafine particles (F1 and F2) is also presented. The particle size ranges of milled AGN powders have already been reported [11]. According to the SEM images of F1 and F2, an irregular shape was observed (S1 Fig). With ultrafine AGN particles (F2) processed by HME, oral AGN-based formulations were prepared according to the composition and processing conditions detailed in Tables 1 and 2. Due to the high surface area to volume ratio of F2 compared to F1, the HME process with ultrafine particles (F2) is expected to produce a more homogeneous dispersion of poorly water-soluble matrix components. Pulverized AGN powders, not in the form of active components of AGN, were put into the HME machine with or without Soluplus. Cytoskeleton components of the AGN root (i.e. celluloses) seem to participate as principal matrices for solid formulations. Throughout the heated barrel, AGN, with or without Soluplus, was processed into homogeneous extrudates by heating and shear stress (dependent on the screw configuration). Melted extrudates were successfully prepared by the described processing conditions and pulverized into powder form for further studies.

**Table 4:** Particle size analysis of AGN powders after milling.

Formulations	Particle size (μm)			
	d(0.1)	d(0.5)	d(0.9)	mean ± SD
**F1**	33.6	276.9	721.1	329.3 ± 2.4
**F2**	7.9	37.9	102.9	47.9 ± 2.5

d(0.1): Particle diameter at 10% of the volume distribution.

d(0.5): Particle diameter at 50% of the volume distribution.

d(0.9): Particle diameter at 90% of the volume distribution.

Average values of d(0.1), d(0.5) and d(0.9) are presented.

### Water absorption studies

Water absorption-related activities of developed AGN-based formulations were evaluated using three parameters: WAI, WS, and SP. As shown in Table 5, WS values of HME-processed formulations (F3-F8) were higher than the milled particles of AGN (F1 and F2). Notably, the higher weight ratio of Soluplus induced an increase in WS values (F5-F8). HME processing with high shear stress and the addition of Soluplus can make more channels responsible for the permeation and penetration of water into the core of matrix. Using SEM images, we observed the formation of multi-pores on the surface of Soluplus-included formulations (F8), which were not observed in the absence of Soluplus in F5 (S2 Fig). In contrast, WAI and SP values were lower in the HME-processed and higher in the Soluplus-included formulations (F3-F8) than the milled particles of AGN (F1 and F2). Reduced WAI values indicate that a greater soluble component of AGN can be obtained by processing AGN with higher shear stress and the addition of Soluplus. Increased WS values in AGN and Soluplus-based groups with HME-processing suggest that poorly water-soluble components were efficiently extracted in the aqueous environment.

**Table 5:** Water absorption-related parameters and particle size analysis of supernatant of the suspension of AGN formulations.

Formulations	Water absorption-related parameters			Particle size of supernatant of the suspension of formulations	
	WAI	WS (%)	SP	Mean diameter (nm)	Polydispersity index
**F1**	7.41 ± 0.50	35.69 ± 0.94	11.52 ± 1.31	506.4 ± 56.4	0.31 ± 0.03
**F2**	4.27 ± 0.41	41.54 ± 1.24	7.31 ± 1.28	676.9 ± 21.7	0.38 ± 0.01
**F3**	4.75 ± 0.42	43.35 ± 1.49	7.65 ± 1.42	664.8 ± 103.1	0.39 ± 0.05
**F4**	4.63 ± 0.93	45.21 ± 1.28	7.61 ± 0.88	443.3 ± 23.5	0.28 ± 0.02
**F5**	4.18 ± 0.83	48.10 ± 1.30	7.08 ± 0.41	495.0 ± 67.9	0.30 ± 0.03
**F6**	3.75 ± 0.08	40.72 ± 0.26	6.33 ± 0.39	434.5 ± 12.8	0.27 ± 0.01
**F7**	2.63 ± 0.06	51.34 ± 1.37	5.34 ± 0.04	177.8 ± 5.9	0.23 ± 0.02
**F8**	2.12 ± 0.20	68.21 ± 0.19	6.65 ± 0.16	182.0 ± 4.6	0.33 ± 0.01

WAI: Water absorption index, WS: water solubility, SP: swelling power.

Data are presented as mean ± SD (n = 3).

### Particle size analysis of the aqueous dispersion of formulations

**Fig 3:**
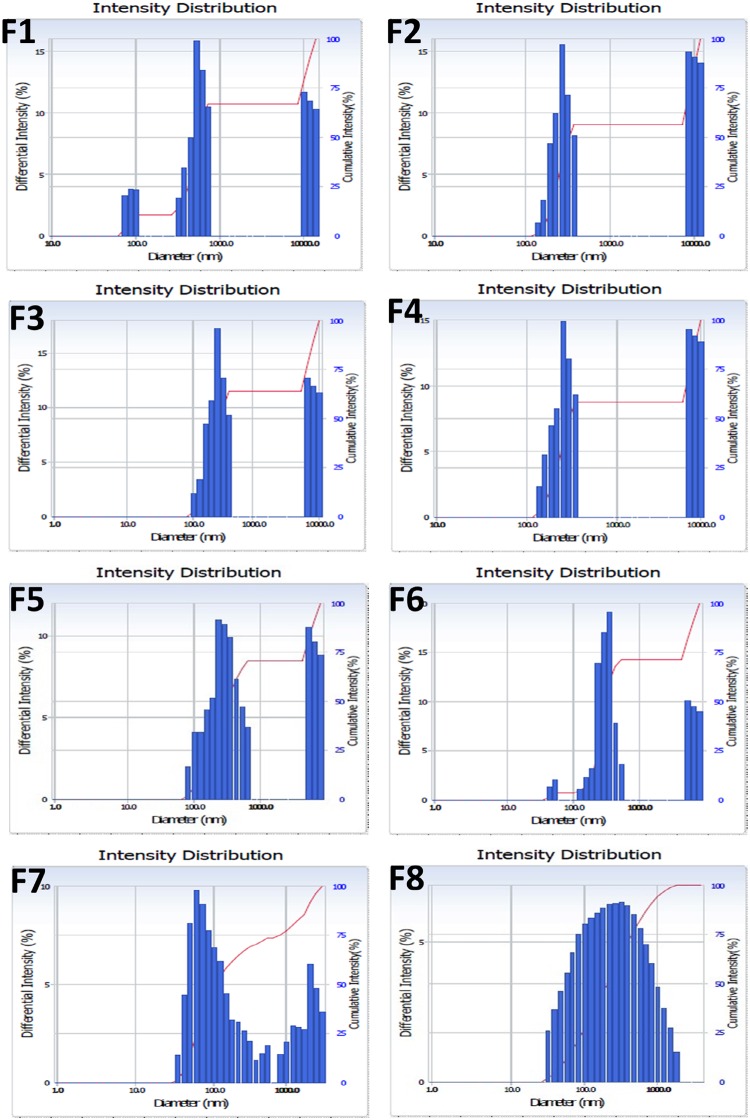
Size distribution of the aqueous dispersion of formulations (F1-F8).

After centrifugation, the supernatant of the aqueous dispersion of developed formulations was obtained, and its particle size and size distribution were measured (Fig 3 and Table 5). Although nano-sized dispersion was observed in all groups, the application of higher shear stress and the addition of Soluplus produced smaller particles (<200 nm). Moreover, the size distribution of that group (F8) was narrower and more uniform (unimodal), than the other groups (Fig 3). Nano-sized (<200 nm) and narrowly distributed particles can contribute to the enhanced intestinal permeation of the active components of AGN. Homogeneous dispersion of the active components of AGN in the matrix, achieved by high shear stress and the addition of Soluplus, may be related to the separation of nano-sized particles from the developed formulations. It is assumed that these nanoparticles are derived from components of AGN and Soluplus after HME processing.

### Extraction test

**Fig 4:**
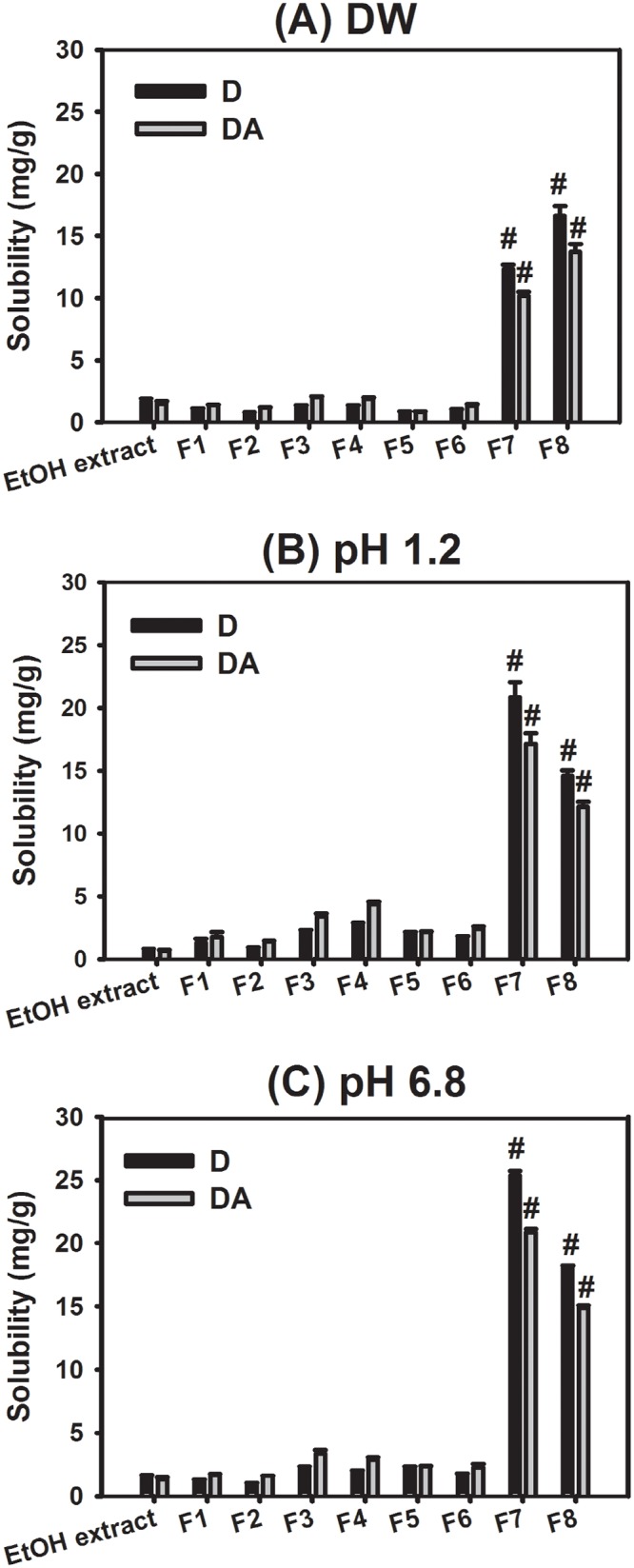
Released amounts (mg/g, w/w) of D and DA in DW (A), pH 1.2 (B), and pH 6.8 (C). Data are presented as mean ± SD (n = 3). ^#^ *p* < 0.05, compared to D or DA in AGN EtOH ext group. Two-tailed *t*-test is used for the statistical analysis.

The amount of D and DA, the active components of AGN, extracted from AGN in water was quantitatively analyzed after 2-h incubation in different media (Fig 4). The solubility of D and DA in AGN EtOH ext, AGN particles (F1 and F2), and HME-processed formulations (F3-F8) was measured in DW, pH 1.2 buffer, and pH 6.8 buffer. The buffers at pH 1.2 and 6.8 simulate gastric fluid and intestinal fluid, respectively. Thus, they can be used to predict the amount of D and DA released in the gastrointestinal tract after oral administration.

The contents of major compounds in the prepared AGN EtOH ext are as follows; 61.00 ± 12.63 mg/g (D); 49.30 ± 12.13 mg/g (DA); 2.19 ± 0.039 mg/g (DOH). The influence of processing temperature of HME on the major components (D and DA) of AGN was also investigated (S3 Fig). Even after 6 h incubation (longer than HME processing period) of AGN EtOH ext at 100°C, the contents (%) of D and DA were maintained around 90%. It implies that the major components of AGN can be preserved in the HME processing condition.

As shown in Fig 4, the solubilities of D and DA in the F7 and F8 groups were significantly higher than those in the AGN EtOH ext group (*p* < 0.05) in all kinds of media (DW, pH 1.2 and 6.8 buffers). The solubilities of D and DA in the AGN EtOH ext in DW were 1.68 ± 0.23 and 1.50 ± 0.20 mg/g (the amount of D or DA per the weight of each sample), respectively. The extraction efficiencies, which can be defined as the ratio (%) between the solubility in the medium and the content included in the EtOH ext, of D and DA in AGN EtOH ext were less than 5% in DW and buffers (pH 1.2 and 6.8). Particularly, the solubilities of D and DA in F8 (AGN:Soluplus = 50:50) in DW were 9.91- and 9.14-fold higher than those in the AGN EtOH ext group, respectively. In both pH 1.2 and 6.8 buffers, the solubilites of D and DA in the F8 group were significantly higher than those in the AGN EtOH ext group. In the F8 group, the values at pH 1.2 were 20.12- and 19.08-fold higher than those in the AGN EtOH ext group. In addition, at pH 6.8, 12.23- and 11.23-fold increase in the solubilities of D and DA in F8 was observed compared to the AGN EtOH ext group. HME processing with high shear stress and the addition of a higher percentage of Soluplus seem to improve the dissolution of the poorly water-soluble components D and DA in this study. This result can be explained by the increased WS value (Table 5). Specifically, Soluplus induced the formation of pores on the surface of the AGN-based matrix, and it seemed to be related to the highly efficient extraction of the active components of AGN in the aqueous milieu (S2 Fig). Its solubility- and dissolution-enhancing effects of poorly water-soluble components were already reported [27,28]. Although the crystalline/amorphous states of the active components of AGN were not demonstrated in this study, the molecular dispersion of the active components in the matrix seemed to occur by melting (induced by the temperature of the barrel) and high shear (generated by screw configuration). The enhanced solubility of D and DA in the F8 group observed in the simulated gastrointestinal fluids could lead to the improvement of their intestinal absorption.

### *In vivo* pharmacokinetic study

**Fig 5:**
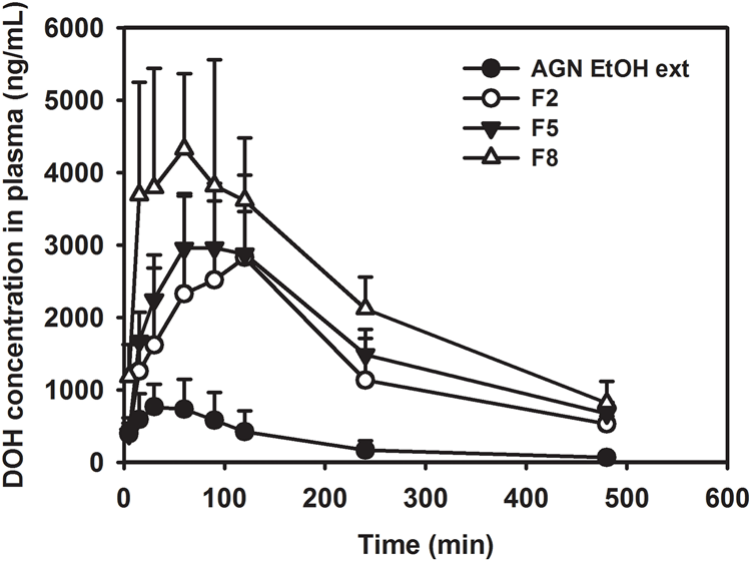
Pharmacokinetic profiles of DOH in plasma after oral administration of AGN EtOH ext, F2, F5, and F8 in rats. Each point indicates means ± SD (n = 3–5).

The pharmacokinetics of DOH was investigated in rats after oral administration of AGN EtOH ext, F2, F5, or F8 (Fig 5 and Table 6). Previous reports have found that orally administered D and DA are metabolized to DOH in the liver [8,29]. Therefore, we evaluated the oral absorption of D and DA by measuring DOH concentration in plasma. DOH content in AGN has been reported to be much lower than both D and DA [2], and data quantitatively analyzed by LC-MS/MS from our preliminary study with AGN EtOH ext confirms this finding. Thus, after oral administration of AGN-based formulations, DOH concentration in plasma can be linked to the extent of D and DA intestinal absorption. It is known that DOH administration has a protective effect against memory impairment in mice [30]. Doses for oral administration were determined based on the equivalent amounts of D and DA in each formulation, as measured by LC-MS/MS. AGN EtOH ext, F2 (ultrafine particle of AGN), F5 (HME product with high shear), and F8 (Soluplus-included HME product with high shear) were used to delineate the influence of ultrafine milling, HME processing, and the addition of Soluplus on the oral absorption of D and DA. As shown in Table 6, the relative fraction absorbed (F_rel_) followed this order: AGN EtOH ext < F2 < F5 < F8. The relative oral bioavailability of DOH in the F8 group was 8.75-fold higher than that of the AGN EtOH ext group. By comparing AUC values between 4 groups, the influence of ultrafine milling, HME processing, and the addition of Soluplus was obvious. C_max_ values of formulations (F2, F5, and F8) were also higher than that of the AGN EtOH ext group. The improved oral bioavailability of F8 can be explained by the enhanced extraction of D and DA in gastrointestinal fluids (Fig 4). As reported [31], the mucosal absorption enhancing effect of Soluplus for poorly water-soluble drugs may also contribute to the improved oral bioavailability of AGN components.

**Table 6:** Pharmacokinetic parameters of DOH after oral administration of AGN EtOH extract, F2, F5, and F8 in rats (n = 3–5).

Parameter	AGN EtOH ext	F2	F5	F8
**AUC (μg∙min/mL)**	151.40 ± 58.29	784.27 ± 174.27^#^	973.14 ± 85.80^#^	1323.45 ± 103.14^#^ ^,^ * ^.^ ^+^
**C**_**max**_ **(ng/mL)**	724.51 ± 340.80	2978.75 ± 1432.02^#^	3090.92 ± 633.51^#^	4605.54 ± 1066.51^#^
**T**_**max**_ **(min)**	60 (30–120)	120 (60–120)	90 (60–120)	90 (30–120)
**F**_**rel**_ **(%)**	100	518	643	875

Data were expressed as means ± SD except for median (ranges) T_max_.

^#^ *p* < 0.05, compared to AGN EtOH ext group.

*p < 0.05, compared to F2 group.

^+^ *p* < 0.05, compared to F5 group.

ANOVA is used for the statistical analysis.

### Histological assay

**Fig 6:**
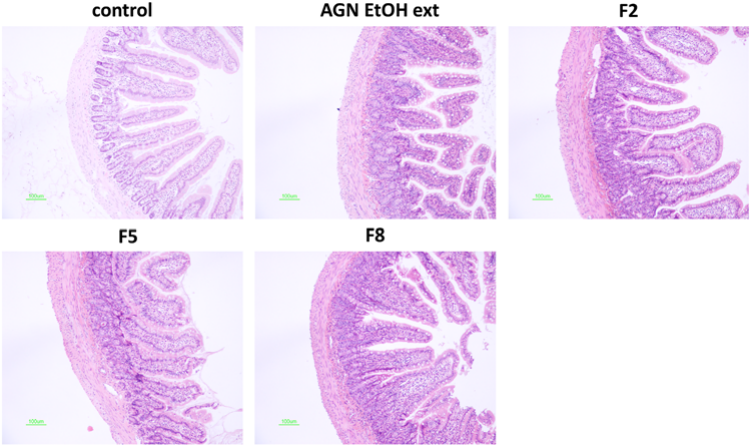
*In vivo* toxicity test in the intestinal epithelium after oral administration of AGN EtOH ext, F2, F5, and F8 in rats. Jejunum of rat was excised at 24 h post-administration of each formulation orally and the tissue was stained by H&E. The length of scale bar (green color) is 100 μm.

The acute toxicity of developed formulations on the intestinal epithelium was evaluated by histological assay (Fig 6). The influence of AGN EtOH ext, F2, F5, and F8 on the intestinal epithelium was assessed by the H&E method. As shown in Fig 6, the application of prepared solid formulations did not induce toxicity on mucosal structures, including microvilli and cell junctions. Pathological changes including inflammation and erosion were also not detected in these groups (F2, F5, and F8). Soluplus, included in the F6-F8 formulations, is a grafted copolymer consisting of polyethylene glycol (PEG) 6000, vinylcaprolactam, and vinyl acetate. Its oral LD_50_ value was higher than 5 g/kg, according to the manufacturer’s data (BASF SE, Ludwigshafen, Germany). Considering the dose of AGN formulations in this study, the amount of Soluplus administered is less than the LD_50_ value. Thus, Soluplus, at the given weight ratio, can be used to make oral solid formulations of AGN by HME processing without severe toxicity. These findings suggest that these orally administered formulations can be used safely.

### *In vivo* pharmacological efficacy test

**Fig 7:**
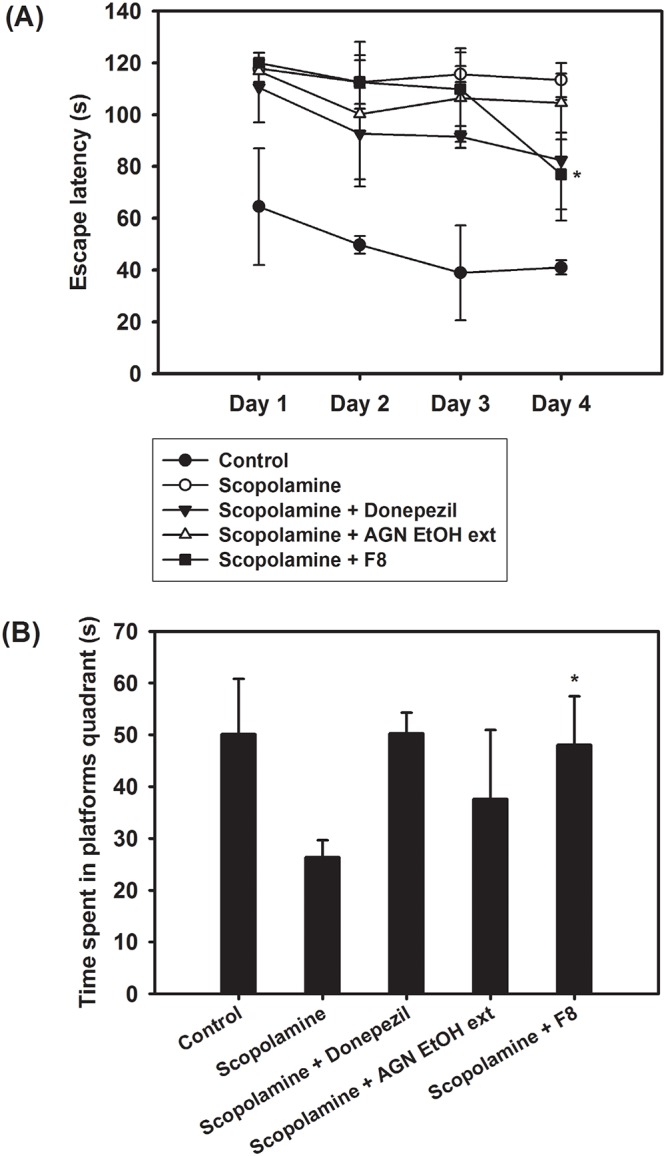
The effect of AGN EtOH ext and F8 on the scopolamine-induced memory impairment in mice. (A) Morris water maze test was done for 4 days after oral administration at a dose of 200 mg/kg (the amount of EtOH ext of AGN per body weight). (B) Time (s) in target quadrant was shown after probe trial test. *p < 0.05, compared to scopolamine-treated group. Each point represents mean ± SD (n = 4–6). Two-tailed *t*-test is used for the statistical analysis.

**Fig 8:**
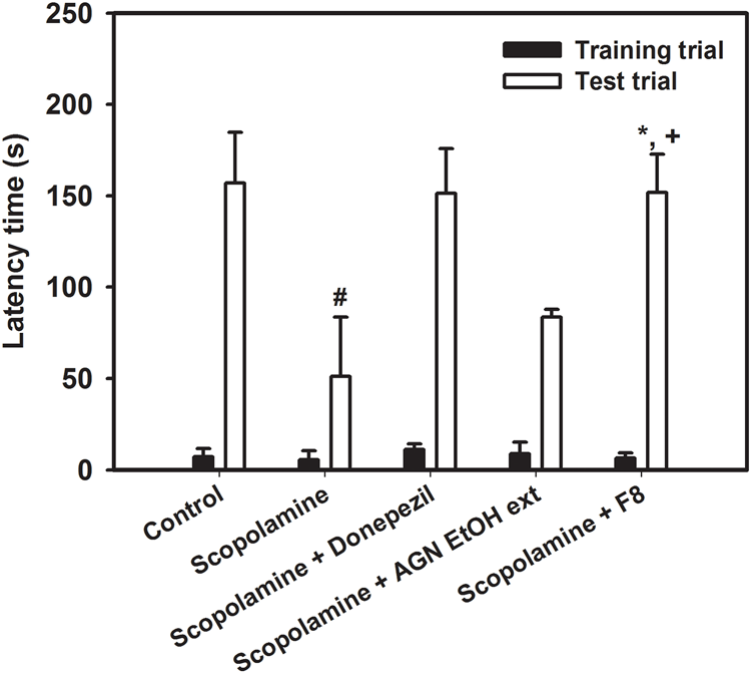
The influence of AGN EtOH ext and F8 on the scopolamine-induced memory impairment mice, evaluated by passive avoidance test. ^#^ *p* < 0.05, compared to control group; *p < 0.05, compared to scopolamine-treated group; ^+^ *p* < 0.05, compared to (scopolamine + AGN EtOH ext)-treated group. Two-tailed *t*-test is used for the statistical analysis. Each point represents mean ± SD (n = 6).

F8 exhibited the highest systemic exposure to DOH after oral administration in our pharmacokinetic study in rats (Fig 5); thus it was selected for further *in vivo* memory-impairment tests along with the EtOH ext of AGN (Figs 7 and 8). The memory enhancing effect of AGN EtOH ext and F8 on scopolamine-induced memory impairment was evaluated by the Morris water maze test and passive avoidance test in mice. Scopolamine is a muscarinic cholinergic receptor antagonist and is known to induce memory dysfunction in behavioral tests.

The Morris water maze test was performed to evaluate hippocampus-dependent spatial memory and learning. In the Morris water maze test, spatial learning and memory were assessed in mice by monitoring the latency to locate the platform. The effect of AGN EtOH ext and F8 on the escape latency was evaluated by the Morris water maze test (Fig 7). The control group exhibited shorter escape latencies during the test trials than the scopolamine-treated group. The scopolamine-treated group maintained a constant latency to escape across 4 days. Donepezil, used as a positive control, had a lower escape latency value for those 4 days than the scopolamine-treated group. Notably, F8 had a comparable escape latency value to that of the donepezil-treated group and a significant reduced escape latency value compared to the scopolamine-treated group (*p* < 0.05). Learning and memory in mice were also assessed by a probe trial test on the last day of the examination (Fig 7B). Mice receiving scopolamine treatment displayed a shorter swimming time in the target quadrant than the control group. After scopolamine treatment, the F8-administered group spent more time in the target quadrant (*p* < 0.05). Moreover, in scopolamine-treated mice, the latency period of the F8-treated group was comparable to that of the donepezil-treated group. These findings suggest that the oral administration of F8 could improve long-term memory.

We performed the passive avoidance test to compare the memory enhancing effect of AGN EtOH ext and F8 (Fig 8). The passive avoidance test was used to evaluate memory based on fear motivation. Latency time was defined as the period required to move from the light to the dark compartment after exposure to foot shock. In the training trial, there was no significant difference in latency time between all groups. This result indicates that drug administration did not influence the training trial. In the test trial, the scopolamine-treated group exhibited a decreased latency to escape compared to the control group (*p* < 0.05). Both the AGN EtOH ext and F8-treated groups displayed an increased latency time in the test trial compared to the scopolamine-treated group. Particularly, the F8-administered group presented a significantly increased latency time compared to scopolamine- and AGN EtOH ext-administered groups (*p* < 0.05).

Considering all these findings, F8 has a more potent memory-enhancing effect than AGN EtOH ext. This result may be due to a greater increase in the AUC value for the F8-treated group than the AGN EtOH ext group (Fig 5 and Table 6). Therefore, this AGN and Soluplus-based formulation processed by HME may be useful as an efficient oral formulation for the treatment of memory impairment.

Oral solid formulations based on AGN and Soluplus were prepared by HME processing. Before HME, AGN was pulverized into particles and their physicochemical properties were investigated. Under HME processing with high shear with the addition of Soluplus, AGN-based extrudates were produced. The extracted amounts of AGN active components (D and DA) from F8 formulation, were increased in simulated gastrointestinal fluids compared to the AGN EtOH ext group. The relative oral bioavailability of DOH in F8-administered rats was 8.75-fold higher than that of the AGN EtOH ext group. In scopolamine-induced memory impaired mice, the cognitive enhancing effect of AGN/Soluplus-based formulation (F8) was significantly greater than the AGN EtOH ext group in the Morris water maze test and passive avoidance test. AGN and Soluplus-based solid formulations developed by HME technique can accomplish the improvement of the aqueous solubility, dissolution, and intestinal absorption of poorly water-soluble components of AGN and subsequent enhancement of memory. HME equipped with twin screw for generating high shear can alter the physicochemical properties of AGN components. Enhanced oral absorption and memory ameliorating effect can contribute to reduce daily intake amounts of AGN. Moreover, versatile processability of extrudates into various dosage forms, such as powder, granule, tablet, and capsule, can increase the usefulness as a dietary supplement. Soluplus-included solid formulation prepared by HME can be a promising carrier for oral delivery of phytochemicals.

## Supporting Information

S1 Fig SEM images of coarse (F1) and ultrafine (F2) AGN powders after milling. The length of scale bar in the image was 20 μm.
(TIF)

S2 Fig SEM images of surface morphology of AGN formulations (F5 and F8). The length of scale bar in the image was 1 μm.
(TIF)

S3 Fig The contents (%) of D and DA in AGN EtOH ext after incubation 6 h at different temperature (20, 60, and 100°C). The percentage of content, compared to that value of AGN EtOH ext stored at -20°C, was presented. Data represent means ± SD (n = 3).
(TIF)

S1 Materials and Methods (DOCX)
