# Supplementary figures and images for: Angelica gigas Nakai and Soluplus-Based Solid Formulations Prepared by Hot-Melting Extrusion: Oral Absorption Enhancing and Memory Ameliorating Effects
Source: PLoS One. 2015 Apr 27;10(4):e0124447. doi: 10.1371/journal.pone.0124447 (PMC4411051; doi:10.1371/journal.pone.0124447)

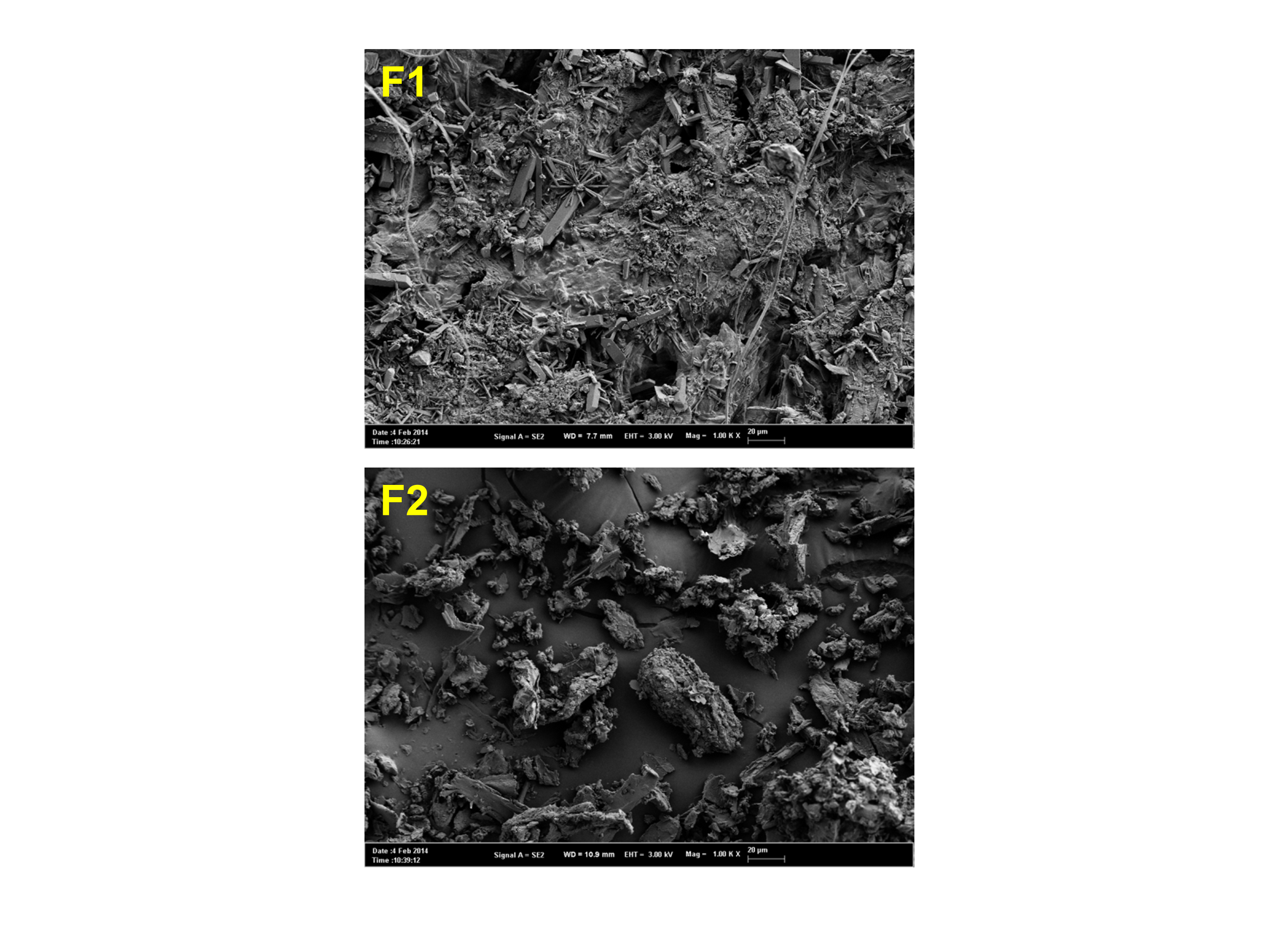

Supplement: S1 Fig — The length of scale bar in the image was 20 μm. (TIF) [file pone.0124447.s001.tif]

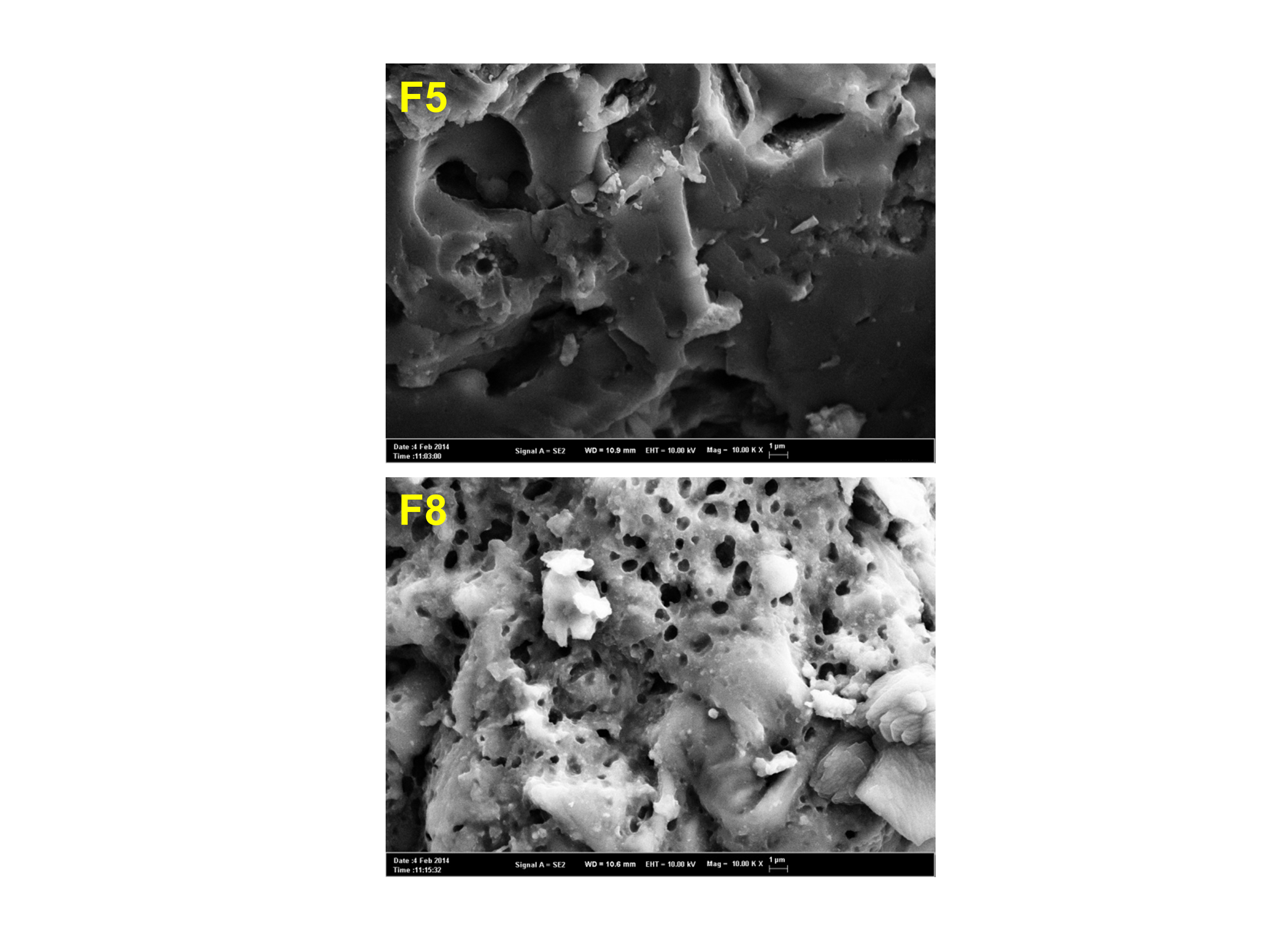

Supplement: S2 Fig — The length of scale bar in the image was 1 μm. (TIF) [file pone.0124447.s002.tif]

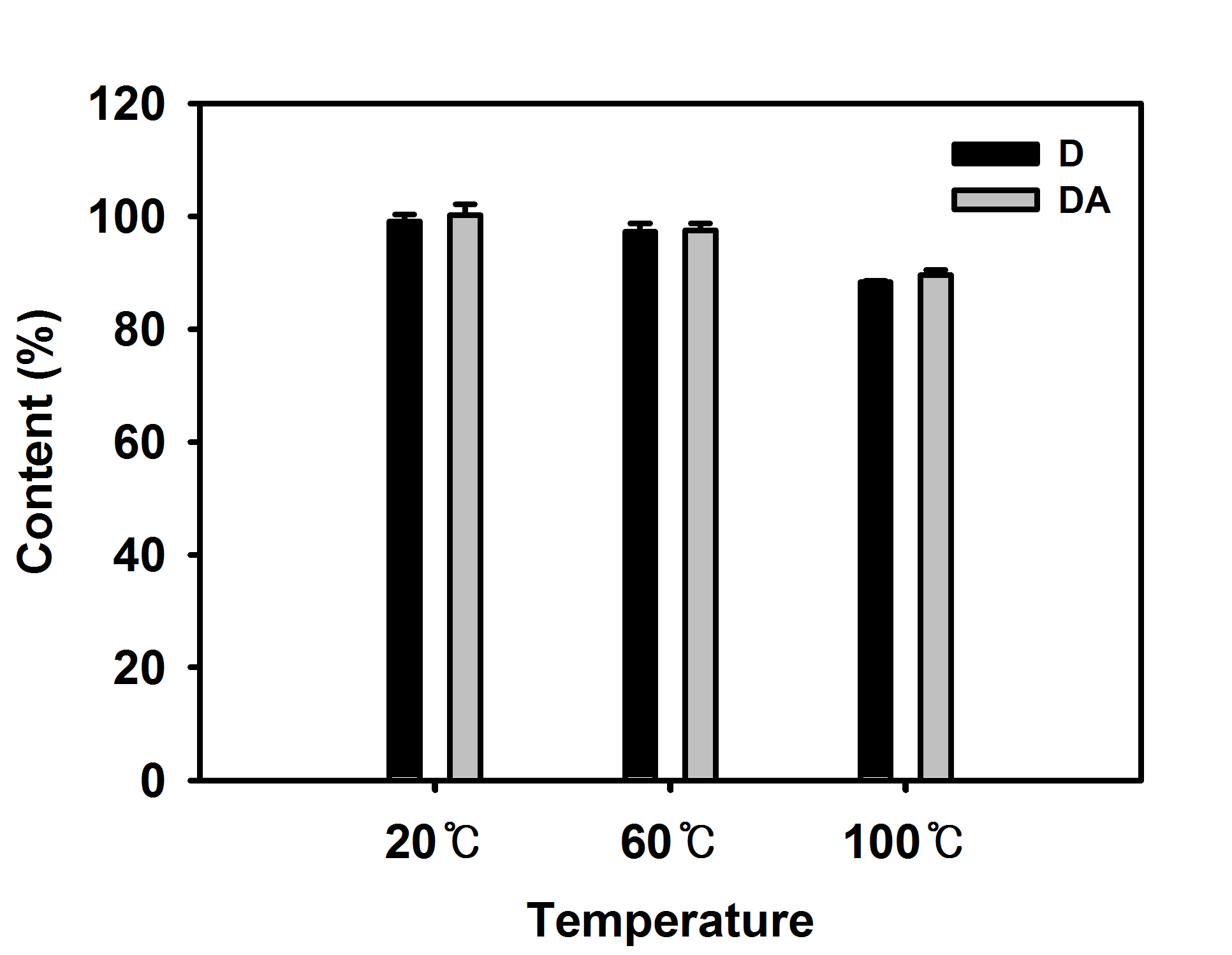

Supplement: S3 Fig — The percentage of content, compared to that value of AGN EtOH ext stored at -20°C, was presented. Data represent means ± SD (n = 3). (TIF) [file pone.0124447.s003.TIF]
