# Supplementary material for: Angelica gigas Nakai and Soluplus-Based Solid Formulations Prepared by Hot-Melting Extrusion: Oral Absorption Enhancing and Memory Ameliorating Effects
Source: PLoS One. 2015 Apr 27;10(4):e0124447. doi: 10.1371/journal.pone.0124447 (PMC4411051; doi:10.1371/journal.pone.0124447)
Supplement: S1 Materials and Methods — (DOCX) [file pone.0124447.s004.docx]

**Materials & Methods**

**Figure S1.**

The morphology of milled powders of AGN (F1 and F2) was characterized by variable pressure field emission scanning electron microscopy (VP-FE-SEM; SUPRA-55VP, Carl Zeiss, Oberkochen, Germany) with an acceleration voltage of 5 kV. Each sample was coated with platinum for 90 s before taking a photograph.

**Figure S2.**

The surface morphology of AGN formulations (F5 and F8) was characterized by variable pressure field emission scanning electron microscopy (VP-FE-SEM; SUPRA-55VP, Carl Zeiss, Oberkochen, Germany) with an acceleration voltage of 5 kV. Each sample was coated with platinum for 90 s before taking a photograph.

**Figure S3.**

AGN EtOH ext was incubated for 6 h at different temperature (20, 60, and 100 ℃). Each sample was dissolved in methanol (2 mg/ml concentration) and diluted with mobile phase for LC-MS/MS method. The contents of D and DA were quantitatively analyzed by described LC-MS/MS method. Content of D or DA is presented as percentage value compared to that value of incubation group at -20℃.
